# The impact of individual and combined abiotic factors on daily otolith growth in a coral reef fish

**DOI:** 10.1038/srep28875

**Published:** 2016-06-28

**Authors:** Amelia S. Wenger, James Whinney, Brett Taylor, Frederieke Kroon

**Affiliations:** 1 ARC Centre of Excellence for Coral Reef Studies, James Cook University, Townsville, QLD, 4811, Australia; 2 College of Science, Technology, and Engineering, James Cook University, Townsville, QLD, 4811, Australia; 3 NOAA Fisheries, 1845 Wasp Boulevard, Building 176, Honolulu, Hawaii, 96818, USA; 4 Australian Institute of Marine Science, Townsville, QLD, 4810, Australia

## Abstract

Coral reefs are increasingly subjected to both local and global stressors, however, there is limited information on how reef organisms respond to their combined effects under natural conditions. This field study examined the growth response of the damselfish *Neopomacentrus bankieri* to the individual and combined effects of multiple abiotic factors. Turbidity, temperature, tidal movement, and wave action were recorded every 10 minutes for four months, after which the daily otolith growth of *N. bankieri* was aligned with corresponding abiotic conditions. Temperature was the only significant driver of daily otolith increment width, with increasing temperatures resulting in decreasing width. Although tidal movement was not a significant driver of increment width by itself, the combined effect of tidal movement and temperature had a greater negative effect on growth than temperature alone. Our results indicate that temperature can drive changes in growth even at very fine scales, and demonstrate that the cumulative impact of abiotic factors can be substantially greater than individual effects. As abiotic factors continue to change in intensity and duration, the combined impacts of them will become increasingly important drivers of physiological and ecological change.

It has been well established that fluctuations in abiotic factors can influence natural environments across multiple organisational scales^1^,^2^,^3^. Abiotic influences on individual species can drive changes in community composition and ultimately ecosystem function^4^,^5^,^6^. As multiple lines of evidence accrue that humans are fundamentally modifying abiotic properties of ecosystems, it has become increasingly imperative to understand how these modifications influence organisms^7^,^8^,^9^.

Coral reefs are one the most productive and biologically diverse marine ecosystems on Earth and also one of the most threatened by human pressure^10^,^11^. Coral reefs are increasingly subjected to local stressors, such as changes in water clarity from land-based runoff^12^ and to global stressors, such as rising temperatures^13^ and decreasing pH levels^14^, due to climate change. Additionally, oceanographic features such as wind-wave climate are changing in response to rising temperatures^15^. Given the changes that are occurring, it is crucial to understand how human-induced and natural fluctuations in abiotic variables interact with each other and how organisms respond to the combined effect of local and global stressors^3^,^16^.

Our understanding of how abiotic factors directly affect coral reef fish has improved substantially over the last several years. Laboratory studies have indicated that turbidity can reduce foraging success, leading to reduced growth^17^. Increased temperature coupled with unlimited food supply can result in higher growth rates of coral reef fish^18^. However, above an optimal temperature, or in low food conditions with high temperature, growth declines^19^. Wave action and water flow can both positively and negatively impact foraging success in planktivorous fishes^20^,^21^. Nevertheless, despite increased knowledge on the effects of single abiotic factors, laboratory studies examining the effects of multiple abiotic factors on fish are in their infancy. Even when they exist, they are often focused on abiotic factors related to climate change, particularly temperature and pH^22^,^23^,^24^. Furthermore, while laboratory studies have the advantage of being able to control multiple abiotic factors, organisms’ responses are often tested under static conditions^24^. In the natural environment, abiotic factors vary over independent spatial and temporal scales. The timing, overlap, and intensity of each abiotic factor will greatly influence the response of individuals^25^. Understanding how abiotic factors influence coral reef fishes under natural conditions remains an important knowledge gap that is fundamental to our understanding of the risks facing marine resources.

Stochastic fluctuations in abiotic factors makes identifying the right scale or appropriate measures to assess the potential effects a challenge. Otolith biochronology is being increasingly employed to hind-cast the effects of abiotic factors on fish, often on annual and decadal scales^26^,^27^,^28^. However, hind-casting over such large time scales often requires the use of biogeochemical proxies or coarse resolution environmental data, both of which increase the uncertainty in identifying specific drivers of change^2^,^29^. A similar approach could be applied to examining the effects of abiotic fluctuations on daily otolith growth in fish. The width of daily otolith increments can provide estimates of daily resolved growth^30^ and has been used and validated as a common proxy for daily growth for several coral reef species^31^,^32^,^33^. While individual variations in growth may exist, the overall shared growth pattern of multiple fish within a population will reflect any environmental signal^34^. Thus, a combination of high resolution turbidity, temperature, wave, and tide (a proxy for water flow) data and daily measurements of otolith growth from fish experiencing known conditions can provide unique insight into how fluctuating environmental conditions may affect fish growth. An analysis of this kind will allow us to refine our understanding of the role of different abiotic factors in driving fish growth and how fish growth may change as each factor changes.

This study examined the daily otolith incremental widths of juvenile *Neopomacentrus bankieri*, a planktivorous damselfish, from three inshore coral reefs in the Great Barrier Reef. Measurements of turbidity, temperature, wave action, and tidal forcing at each reef were taken every 10 minutes for four months prior to the collection of fish. The growth increments of the otoliths of collected individuals were matched with the corresponding daily average of each parameter to determine the strength and significance of turbidity, temperature, tidal fluctuations, and wave action on daily growth.

## Results

### Measurement of environmental parameters

Turbidity ranged from 0-120.8 NTU across all sites from April 2^nd^ to August 2^nd^ in 2013 (Table 1) with peaks usually lasting for a few days before returning to background levels. Temperature ranged from 20.3–30.0 °C across all sites, with daily average temperature reducing throughout the study period from the highest level in April to the lowest in August. Wave action, measured as root mean squared (RMS) pressure, was quite variable throughout the study period, ranging from 0–0.162 m (an RMS of 0.162 m is approximately equivalent to a significant wave height of 1.6 m^35^). The tidal range in the region ranged from 0.9–6.3 m, with variations occurring over a period of approximately 2 weeks (Table 1). Daily average turbidity, wave action, and tidal range varied throughout the study period, whereas daily average temperature had a clear temporal signature (Supplementary Figs S1–4).

### Relationship between somatic growth and otolith growth

There was a strong positively correlated relationship between both otolith length and width and standard length (r^2^ = 0.91 and 0.87, respectively, Supplementary Fig. S5). The residual plots of the model (Supplementary Fig. S6) indicated that the residuals were consistent with stochastic error. There was a much stronger relationship between otolith morphometrics and fish standard length than between the age of the individual and standard length (r^2^ = 0.71).

### Relationship between abiotic factors and daily growth rate

**Figure 1:**
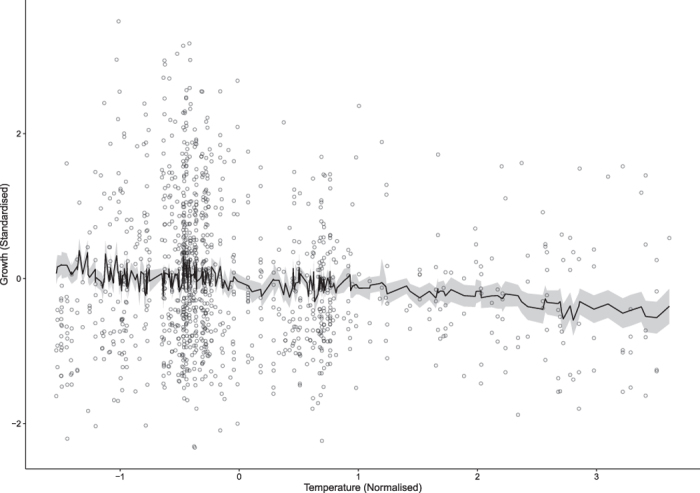
The linear mixed effects model predicted fit of the relationship between normalised temperature and daily otolith increment width. Grey shading around the black line represent bootstrapped 95% confidence intervals. Grey dots represent the raw data.

Temperature was the only abiotic factor that significantly drove changes in growth, with higher temperatures leading to lower growth rates (P = 0.0013; Table 2; Fig. 1). This was regardless of the time of year the fish were caught, as date of growth was included in the model. Although not significant, there was a negative relationship between otolith growth and tidal range (Supplementary Fig. S7). Conversely, there was a positive, but non-significant relationship between wave action and otolith growth (Supplementary Fig. S8). Finally, there was no trend between turbidity and growth (Supplementary Fig. S9).

### Effect of cumulative impacts on growth

**Figure 2:**
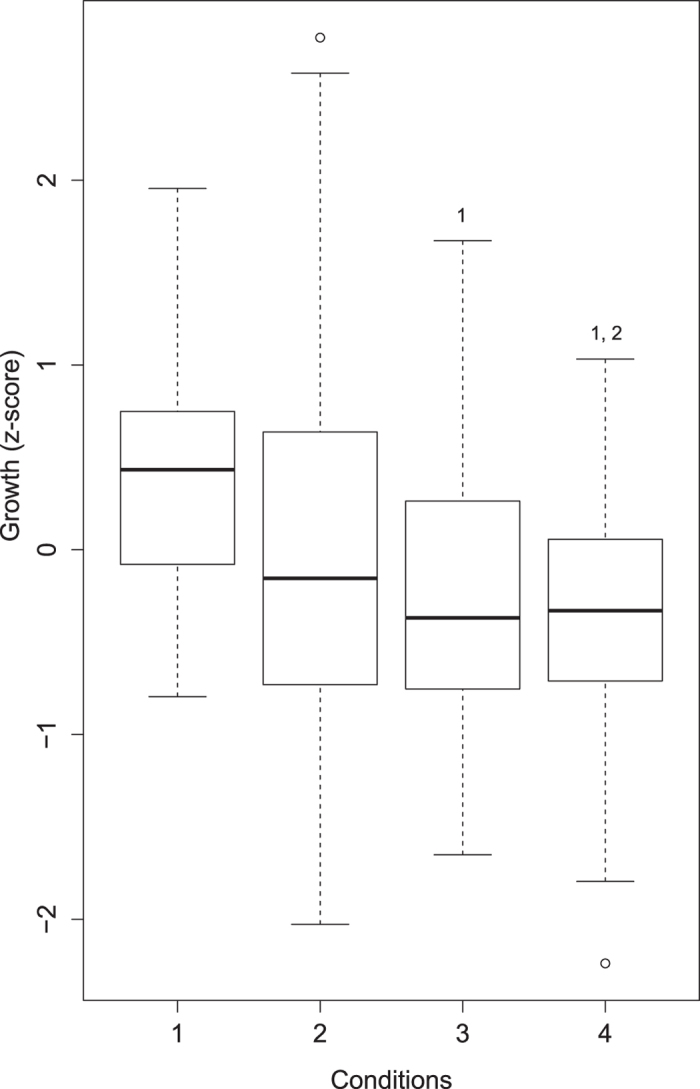
Box plot of the individual and combined effects of temperature and tidal range. Condition 1 = low temperature, small tidal range (control); 2 = low temperature, large tidal range (individual effect of tides); 3 = high temperature, small tidal range (individual effect of temperature); 4 = high temperature, large tidal range (combined effects of temperature and tide). Numbers above bars indicate the conditions between which there is a significant difference.

The results of the cumulative impact assessment for turbidity and temperature indicated there was no difference in growth among “control”, “individual effects”, or “combined effects” conditions. Growth was significantly lower when fish were exposed to both high temperature only (P = 0.007; Fig. 2) as well as high temperatures and large tidal ranges (P = 0.001; Fig. 2) compared to growth during low temperatures and small tidal range conditions. Growth in fish exposed to the combined effects of high temperature and large tidal ranges was also significantly lower than growth in fish exposed to low temperatures but large tidal ranges (i.e., the individual effect of tide) (P = 0.03). Although there was not a significant difference between the individual effect of temperature and the combined effects of temperature and tide, the Cohen’s *d* values for the effect sizes among the four conditions indicate that the combined effect of temperature and tidal range was greater than the individual effect of each abiotic factor (Table 3).

**Figure 3:**
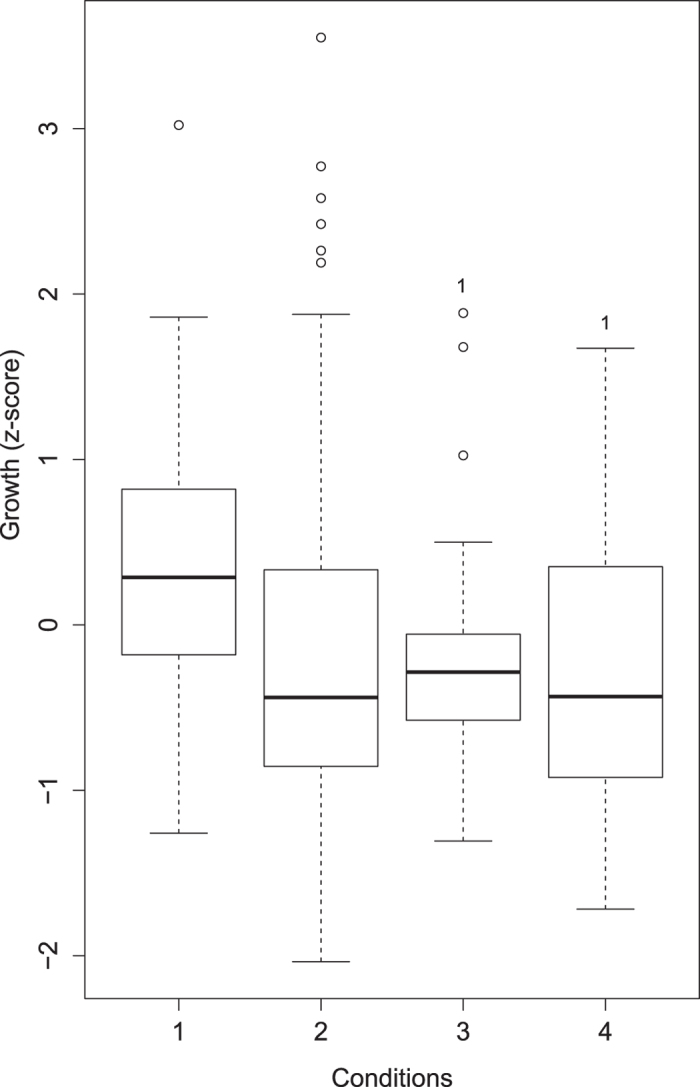
Box plot of the individual and combined effects of temperature and wave action. Condition 1 = low temperature, high wave action (control); 2 = low temperature, low wave action (individual effect of waves); 3 = high temperature, high wave action (individual effect of temperature); 4 = high temperature, low wave action (combined effects of temperature and wave action). Numbers above bars indicate the conditions between which there is a significant difference.

When the combined effects of temperature and wave action were examined, there was a significant reduction in growth between the control condition (low temperature high wave action) and both the combined effects condition (high temperature low wave action) and the individual effect of temperature condition (high temperature high wave action) (P = 0.03; Fig. 3). The Cohen’s *d* values indicate that there was limited additional influence of wave action when combined with temperature (Table 3).

## Discussion

Small scale ecological processes such as foraging and growth are imperative for ecosystem functioning and population persistence^36^,^37^. This study illustrated that temperature was the primary abiotic factor driving coral reef fish otolith growth. Furthermore, our results indicate that even when tidal movement did not individually mediate changes in otolith growth, when combined with temperature, it caused a significant reduction in daily otolith growth beyond those seen by temperature alone. To our knowledge, this is the first time that otolith biochronology has been used to assess how multiple abiotic factors mediate fine-scale changes in coral reef fish otolith growth and represents a significant progression in our ability to detect and predict how abiotic fluctuations impact coral reef fish.

Our results are consistent with previous studies that have also indicated that temperature can mediate otolith growth^27^,^38^. Temperature plays a role in growth due to the acceleration of metabolic rates in warmer temperatures^39^. McLeod *et al*.^18^ found that larvae fed *ad libitum* increased daily growth as temperature increased, but also found that larvae on restricted diets had slower daily growth rates as temperature increased. Faster growth rates in higher temperatures can only be supported if ingestion rates increase, due to increased metabolic rates, which increases exponentially rather than linearly with temperature^40^,^41^. Additionally, increased temperature will only support increased growth up to an optimal temperature, after which, growth rates decline dramatically^42^. McLeod *et al*.^33^ recorded a non-linear relationship between larval otolith growth and mean water temperatures in two species, with the thermal optima for growth being surpassed at low latitude sites. Similarly, Morrongiello and Thresher^38^ only found a strong negative correlation between temperature and otolith growth in tiger flathead when at their equatorward range limit. Given that the results of the present study found a linear reduction in otolith growth as temperature increased, it is possible that there was not more food available to match the demands of the increases in metabolic rate due to increased temperature.

Several studies have identified potential drivers that could influence both otolith increment widths and somatic growth, including feeding regime^43^ and lipid reserves^44^. In contrast, Kingsford *et al*.^45^ found that water chemistry could change increment width, which would be unlikely to drive changes in somatic growth. However, the strong relationship found in this study between otolith dimensions and standard length of individual *N. bankieri* suggests that abiotic factors that are influencing otolith growth will also influence somatic growth. Additional factors, such as carry-over effects from pre-settlement life stages, may influence growth trajectories if they differ consistently among sites^46^. The vast majority of sampled fish in the present study were over 30 days old at the time of capture, thus making it problematic to test for site-specific carry-over effects influencing larval phenotype. However, while individual anomalies may make it difficult to compare otolith increment growth to absolute somatic growth, the use of population level otolith growth as a proxy for population level somatic growth can provide reasonable estimates, particularly in data poor regions or with species where laboratory testing is not possible^29^,^34^.

Previous meta-analyses have examined the potential for additive, synergistic, or antagonistic responses to multiple stressors^16^,^24^. However, all of these meta-analyses were based on experimental studies that were able to control conditions. Given that abiotic variables vary independently, it was not possible in our dataset to completely control for each variable. However, our results show that even when tide did not drive significant variation in growth, the combined effect of tide and temperature was greater than temperature alone. Increased temperature has been shown to reduce the aerobic scope of coral reef fishes, which could diminish their ability to effectively capture prey. Additionally, large tidal fluctuations can increase flow rates, which can make it harder for fish to catch prey^21^. A reduced capacity to react with fast moving prey could increase evasion success in planktonic prey^21^,^47^,^48^. In coral reef fishes, like most other organisms, food acquisition is one of the key daily activities dictating individual performance such as growth, reproduction and life expectancy^49^,^50^. Ultimately foraging success and growth can strongly affect patterns of distribution, abundance and population dynamics^51^.

Contrary to expectations, turbidity had no effect on daily growth. This is in contrast to published studies that show an effect of suspended sediment on coral reef fishes^17^,^21^. One of the potential confounding effects is that the sediment on nearshore reefs in the Great Barrier Reef is nutrient enriched^52^. When sediment is re-suspended, even if the fish could have reduced visual acuity, the nutrient enriched sediment may increase their food supply, and counteract any negative effects on foraging. However, Johansen and Jones^21^ found that *Neopomacentrus bankieri* did not experience a negative effect on foraging until 8 NTU; a daily average exceeded 30% of the time on the study reefs. *N. bankieri* is only found on nearshore reefs, whose communities can possess inherent resistance to higher turbidity based on natural turbidity regimes^53^,^54^. It was not possible to catch a species that occurs across multiple turbidity regimes, due to the extremely low abundances of other species on these coral reefs, despite suitable habitat (A. Wenger, unpublished data). Further research should focus on other species found on both turbid and clear-water coral reefs.

Climate-change models predict that tropical sea surface temperatures will increase by up to 3 °C this century^55^. Our results show that present day temperatures are already negatively affecting growth. While we were not able to measure food availability, food is rarely unlimited in the marine environment. It is evident, based on the results, that *N. bankieri* individuals were not able to maintain consistent growth rates through increases in their food intake. Elevated ocean temperatures are predicted to cause a 2–20% reduction in global marine primary production by 2100 56, which will be superimposed onto plankton communities that are naturally variable on a broad range of spatial and temporal scales^57^. Food variability combined with fluctuating abiotic factors will create a gradient of conditions that fish will face. Planktivorous coral reef fishes play a principal role in the continued health and diversity of coral reef ecosystems. Planktivorous fishes represent ~22% of all coral reef fish species and account for ~60% of the total fish biomass on coral reefs^58^. They are also the main food source for many ecologically and commercially important predator species^59^. Given that fishes in early life history stages require more energy than adults to withstand starvation (due to high metabolic rates and low energy storage) and are more prone to mortality^60^, temperature will differentially affect early life history stages of coral reef fishes. Small changes in mortality during early life history stages can have large impacts on cohort success^61^. Our study highlights the importance of examining systems holistically to be able to truly understand how each variable influences growth.

## Methods

### Measurement of environmental parameters

**Figure 4:**
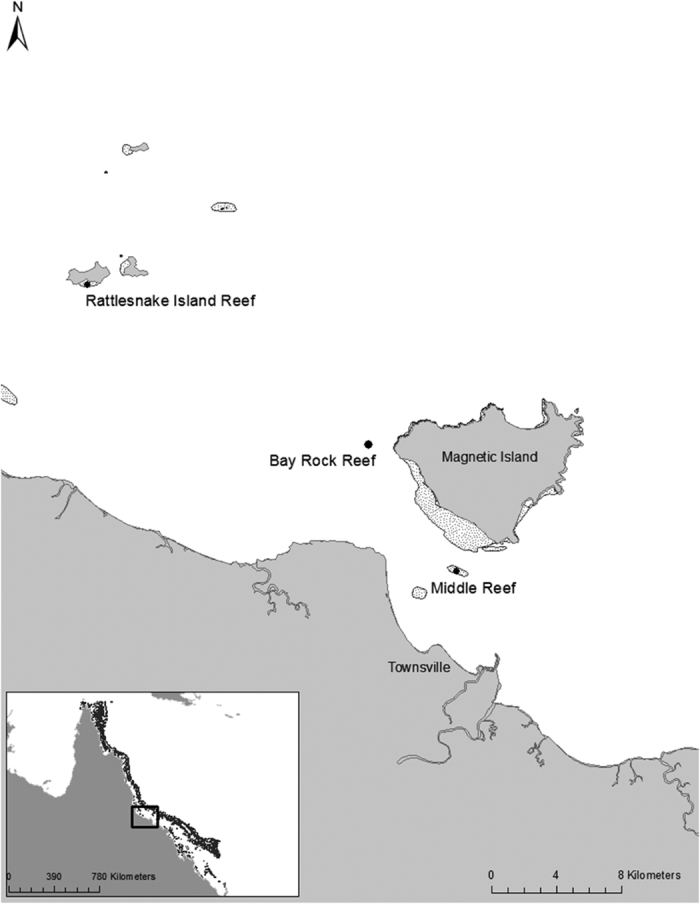
Map of study region. The map was generated using ArcMap v.10.2.1 (desktop.arcgis.com/en/arcmap/).

Three inshore coral reef locations in the Great Barrier Reef were chosen as study sites: Bay Rock Reef, Middle Reef, and Rattlesnake Island Reef (Fig. 4). In the GBR, the term ‘inshore’ applies to areas within 6 to 20 km of the coast^62^,^63^. Ecosystems within this inshore area, including coral reefs, are under pressure from increased sediment and nutrient loads carried by land runoff^64^. The three reefs considered in this study are exposed to runoff from the Burdekin River, the main sources of terrestrially sourced suspended sediment in the GBR^65^.

On the 2^nd^ and 3^rd^ of April, 2013, two nephelometers were placed at each reef within 200 meters of each other. The nephelometers were mounted on heavy steel frames that raised the instrument ~40 cm off the seafloor. Turbidity, temperature and pressure measurements were recorded every 10 min. Each turbidity and temperature record was an average of 250 measurements taken over a 1 sec period, the same was done for pressure; however, 10 consecutive readings were taken over a period of 10 seconds. The mean of the 10 pressure readings was then calibrated to provide a water depth, which was used to measure tidal variation, whilst their root-mean-square was used to give an expression of the variation in seabed pressure due to wave action (in meters). It should be noted that the 10 second period for the pressure measurements may not be long enough to detect all long wavelength swell waves, however these waves are uncommon in the Great Barrier Reef Lagoon. Sensors were equipped with an anti-fouling wiper which was activated every 2 h^66^. The nephelometer was calibrated before deployment to the standard 200 Nephelometer Turbidity Units (NTU). On the 14^th^ of June, each nephelometer was retrieved, the data were downloaded, and the batteries were changed. The nephelometers were then re-deployed in the same locations.

### Fish growth analysis

All collections were approved by the James Cook University Animal Ethics Committee, approval number A1932 and were completed in accordance to the guidelines laid out by the ethics committee and the Great Barrier Reef Marine Park Authority. From July 31^st^-August 2^nd^, 2013, juvenile *Neopomacentrus bankieri* were collected from each reef, between the two nephelometers, using clove oil and hand nets. *Neopomacentrus bankieri* is a planktivorous damselfish primarily found on inshore coral reefs^21^. Thirty-seven, 28, and 21 individuals were collected from Bay Rock, Middle Reef, and Rattlesnake, respectively (Fig. 1). Sagittal otoliths were extracted from each specimen and processed for interpretation of daily growth increments (DGIs). Sagittae were embedded on the end of a glass slide using Crystalbond 509 and ground to the nucleus using a lapidary grinding wheel (1200 grit). Sagittae were then re-affixed to the slide with the ground surface down and polished from the opposite side to produce a transverse section approximately 150 μm thick. Both sides of the resultant transverse section were then polished using 9, 3, and 0.3 μm lapping film sequentially, and polishing ceased when optimum clarity was achieved for interpretation of DGIs. Age was assigned to individuals by counting the DGIs from the core on three independent occasions using a compound microscope and final age was taken as the mean of the three counts, provided all counts were within 10% of the median. Samples with counts >10% of the median were excluded from the analysis. Settlement marks (representing settlement onto the reef benthos and metamorphosis from larval to benthic-associated stages) were identified as Type 1 following^67^.

Increment-width profiles were established for each individual using the Leica IM50 software. Increment widths were measured along the longest axis on the ventral side of the otolith. Increment-width profiles were “transition-centred” following^68^.

### Relationship between somatic growth and otolith growth

In order to assess the relationship between otolith growth and somatic growth, a series of linear regressions were performed. The following relationships were examined: otolith length to standard length, otolith width to standard length, and post-settlement age to standard length. The residual plots of each model were examined to confirm random distribution of residuals.

### Relationship between abiotic factors and daily growth rate

In order to determine the relationship between abiotic factors and daily growth rate, the daily average for turbidity, temperature, wave action, and tidal range was calculated for each reef, by averaging the measurements from each nephelometer. The presence of a clear settlement mark on the otoliths allowed for a calculation of date of settlement by back calculating from their death date using daily otolith rings. The otolith increment growth data for each fish was matched up with the appropriate daily average of the abiotic data. We offset the abiotic data by one day because of the lag time of 24 hours based on previous research which has shown that it takes 24 hours for settlement age pomacentrids to assimilate food and grow^69^. Only the first 14 days post-settlement were used, as this was the most reliable area on the sagittae to age^26^ and the majority of fish (99%) had data spanning this range.

Linear mixed effects modelling fit by restricted maximum likelihood was used to assess the significance of turbidity, temperature, tidal movement, and wave action in explaining variations in growth^38^. Since regression-based models can be sensitive to variables that are correlated, the variance inflation factors (VIF) for all predictor (i.e., abiotic) parameters used in the model were calculated to check for multi-collinearity. The VIFs for all parameters fell well below the common threshold value and therefore, no parameters needed to be excluded on the basis of collinearity^70^. Individual predictors were mean-centred to facilitate model convergence^38^. Because daily growth increments decline with each day, and to ensure the population level trend was not outweighed by individual variability, a standardised daily growth index for each age was calculated as


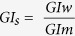


where GI_s_ is the standardised growth, GI_w_, is the individual growth increment width, and GI_m_ is the mean growth increment width within each age group^34^. The linear mixed effects model was generated using the lmer function in the R package lme4^71^, with turbidity, temperature, tide, and wave action set as fixed factors and site and date set as random effects. We assumed a Gaussian distribution and checked the normal distribution of model residuals to confirm goodness of fit. To ensure we were meeting the assumptions of the model, we also checked the plotted residuals to ensure homoscedasticity prior to utilising the results of the model. Final model selection (to obtain the best-fit model while maintaining model parsimony) was decided using Bayesian Information Criterion (BIC)^72^. The significance of each parameter in explaining variation in growth was tested by undertaking Markov Chain Monte Carlo sampling with the function MCMCregress in the R package MCMCpack^73^. Three samples were run using non-overlapping, randomly selected seeds. Chain lengths were set to 1000 with a burnin of 100. A thinning rate of 5 was set to reduce autocorrelation. All chains were combined and chain mixing was tested. Finally, the posterior distribution of the chains was examined to determine the likelihood that the predictor variables were significantly influencing the variation in growth.

### Effect of cumulative impacts on growth

Previous meta-analyses that have examined cumulative impacts have calculated the difference in the effect of individual variables on the response variable and the effect of combined variables, to test for additive, antagonistic, and synergistic effects^16^,^24^. The predicted relationship between each abiotic variable and growth from the linear mixed effects models were used to examine potential cumulative impacts. The 25^th^ and 75^th^ percentiles were calculated for each abiotic factor and only values below and above these percentiles were used. The percentile from both variables that was predicted to result in the highest growth were used as the “control condition”. To test for individual effects of each variable, one predictor variable at a time was changed to the reverse quantile (corresponding to predicted minimum growth) while keeping the other one constant and the corresponding growth data was extracted (individual effects conditions). Finally, to test for combined effects, both variables were changed to the quantile expected to give the minimum growth (combined effects condition). The differences in growth among the conditions were determined using a one way permutation test based on 10,000 Monte-Carlo re-samplings followed by a pairwise permutation test with an adjusted p value generated, both within the “coin” package in R^74^. To determine effect sizes, a Cohen’s *d* for each condition compared to the control was calculated^75^. The Cohen’s *d* value for each “individual effects” condition were combined and compared to the Cohen’s *d* value of the “combined effects” condition. If the values were equal, cumulative impacts would be additive, if the “combined effects” value was greater than the combined “individual effects” values, cumulative impacts would be synergistic, but it was less than the combined “individual effects” the cumulative impacts would be antagonistic. All statistical analyses were performed with R v.3.2.3 (R Core Team 2015).

## Additional Information

**How to cite this article**: Wenger, A. S. *et al*. The impact of individual and combined abiotic factors on daily otolith growth in a coral reef fish. *Sci. Rep.* **6**, 28875; doi: 10.1038/srep28875 (2016).

## Supplementary Material

Supplementary Information

## Figures and Tables

**Table 1:** Summary statistics of turbidity, temperature, wave action, and tidal range across all sites.

		Average	Median	Maximum	Minimum
Bay Rock Reef	Turbidity (NTU)	10.1	6.6	120.8	0.0
	Temperature (C)	24.3	23.7	28.2	21.6
	Wave Action (RMS)	0.03	0.02	0.16	0.001
	Tidal Range (m)	2.3	2.3	3.9	0.9
Middle Reef	Turbidity	7.4	3.5	66.9	0.3
	Temperature	23.6	22.8	30.0	20.6
	Wave Action	0.01	0.01	0.12	0.00
	Tidal Range	2.8	2.7	4.4	1.1
Rattlesnake Island Reef	Turbidity	3.1	1.9	22.7	0.1
	Temperature	23.7	23.0	28.2	20.3
	Wave Action	0.02	0.02	0.13	0.00
	Tidal Range	4.2	4.1	6.3	2.6

**Table 2:** The results from the linear mixed effects models.

Fixed effects	Estimate	Standard Error	df	t value	Pr (>|t|)
Initial linear mixed effects model (BIC = 3288.63)					
Turbidity	−0.05191	0.04608	385	−1.127	0.73998
Temperature	−0.07453	0.03256	248	−3.02	0.0026
Tide	0.01496	0.04745	74	0.315	0.71354
Waves	0.05741	0.04795	176	1.197	0.22490
Final linear mixed effects model (BIC = 3255.75)					
Temperature	−0.07237	0.03106	262	−3.225	0.00197

The lmer function automatically calculates t-tests using Satterthwaite approximations to degrees of freedom.

**Table 3:** The Cohen’s *d* values for the combined effects of temperature and tide and temperature and waves.

	Cohen’s *d*(groups compared to control)
Temperature and tide effects	
low temperature low tide (control)	
low temperature high tide (individual effect of tidal movement)	0.58
high temperature low tide (individual effect of temperature)	1.19
high temperature high tide (combined effects)	1.54
Temperature and wave effects	
low temperature high wave action (control)	
low temperature low wave action (individual effect of waves)	0.79
high temperature high wave action (individual effect of temperature)	1.10
high temperature low wave action (combined effects)	1.07

The sum of the individual effects of temperature and tide is greater than the individual effect of temperature alone, so the cumulative effects of temperature and tide is greater than temperature alone.
