# Supplementary material for: The impact of individual and combined abiotic factors on daily otolith growth in a coral reef fish
Source: Sci Rep. 2016 Jun 28;6:28875. doi: 10.1038/srep28875 (PMC4924089; doi:10.1038/srep28875)
Supplement: Supplementary Information [file srep28875-s1.pdf]

The impact of individual and combined abiotic factors on daily otolith growth in a coral reef fish

Supplementary material

Amelia S. Wenger[1], James Whinney[2], Brett Taylor[3], Frederieke J. Kroon[4]

[1]ARC Centre of Excellence for Coral Reef Studies, James Cook University, Townsville, QLD, Australia 4811

[2] College of Science, Technology, and Engineering, James Cook University, Townsville, QLD, Australia 4811

[3] NOAA Fisheries, 1845 Wasp Boulevard, Building 176, Honolulu, Hawaii, USA, 96818

[4] Australian Institute of Marine Science, Townsville, QLD, Australia, 4810

Author Contact information:

*Amelia S. Wenger: amelia.wenger@gmail.com

James Whinney: james.whinney@jcu.edu.au

Brett Taylor: brett.taylor@noaa.gov

Frederieke Kroon: f.kroon@aims.gov.au

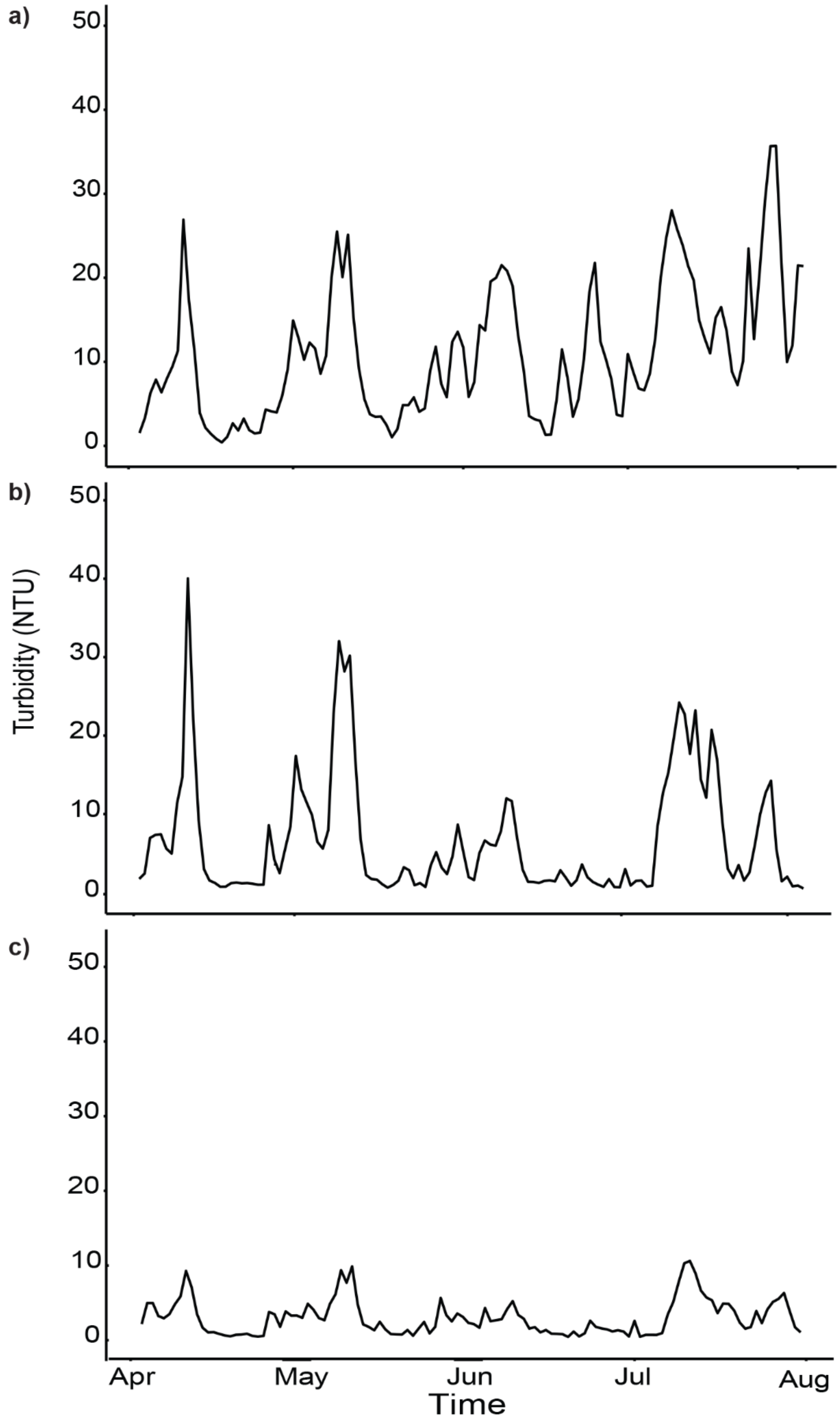

Figure S1: The daily average turbidity recorded at a) Bay Rock Reef, b) Middle Reef, and c) Rattlesnake Island Reef.

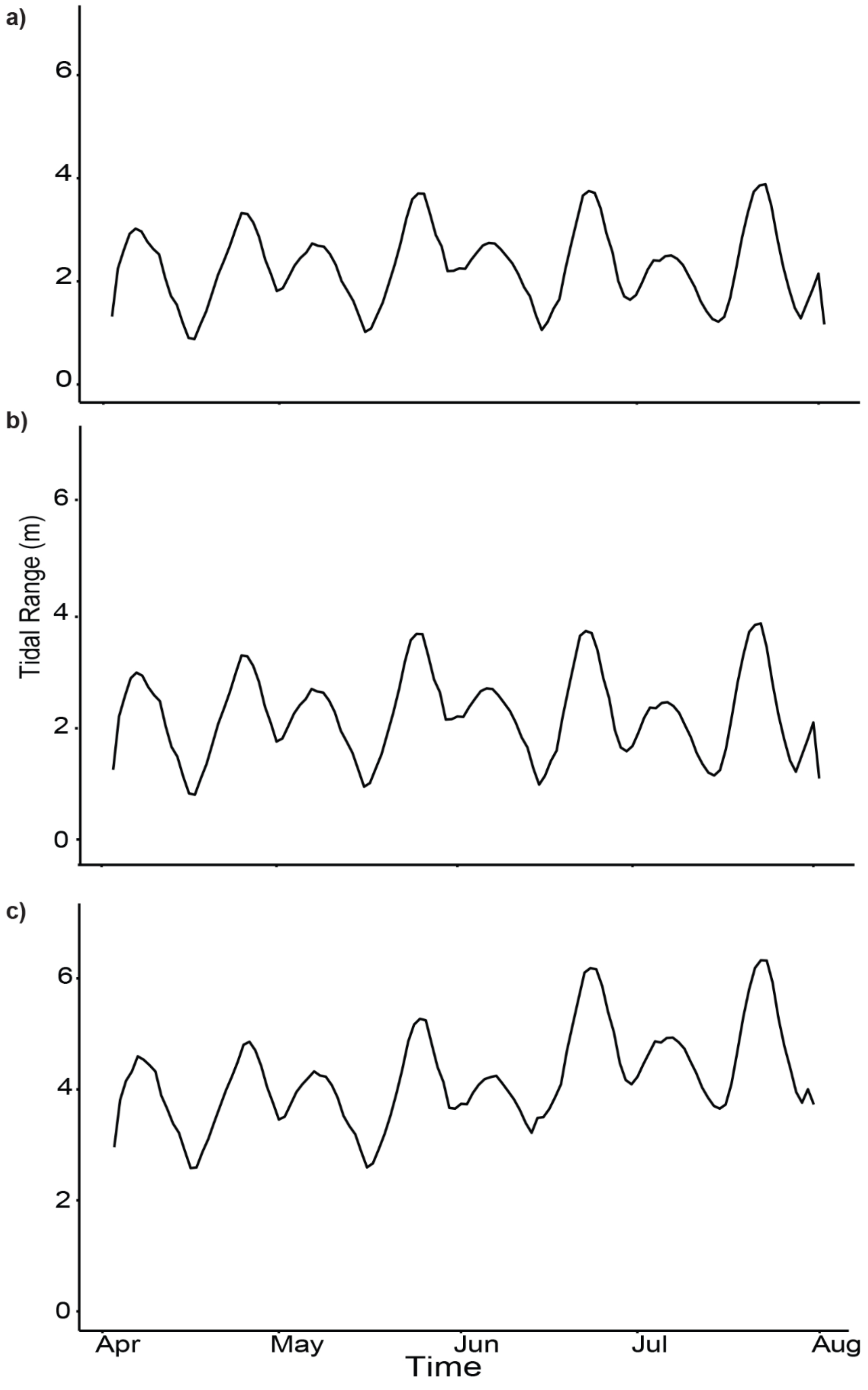

Figure S2: The daily tidal range recorded at a) Bay Rock Reef, b) Middle Reef, and c) Rattlesnake Island Reef.

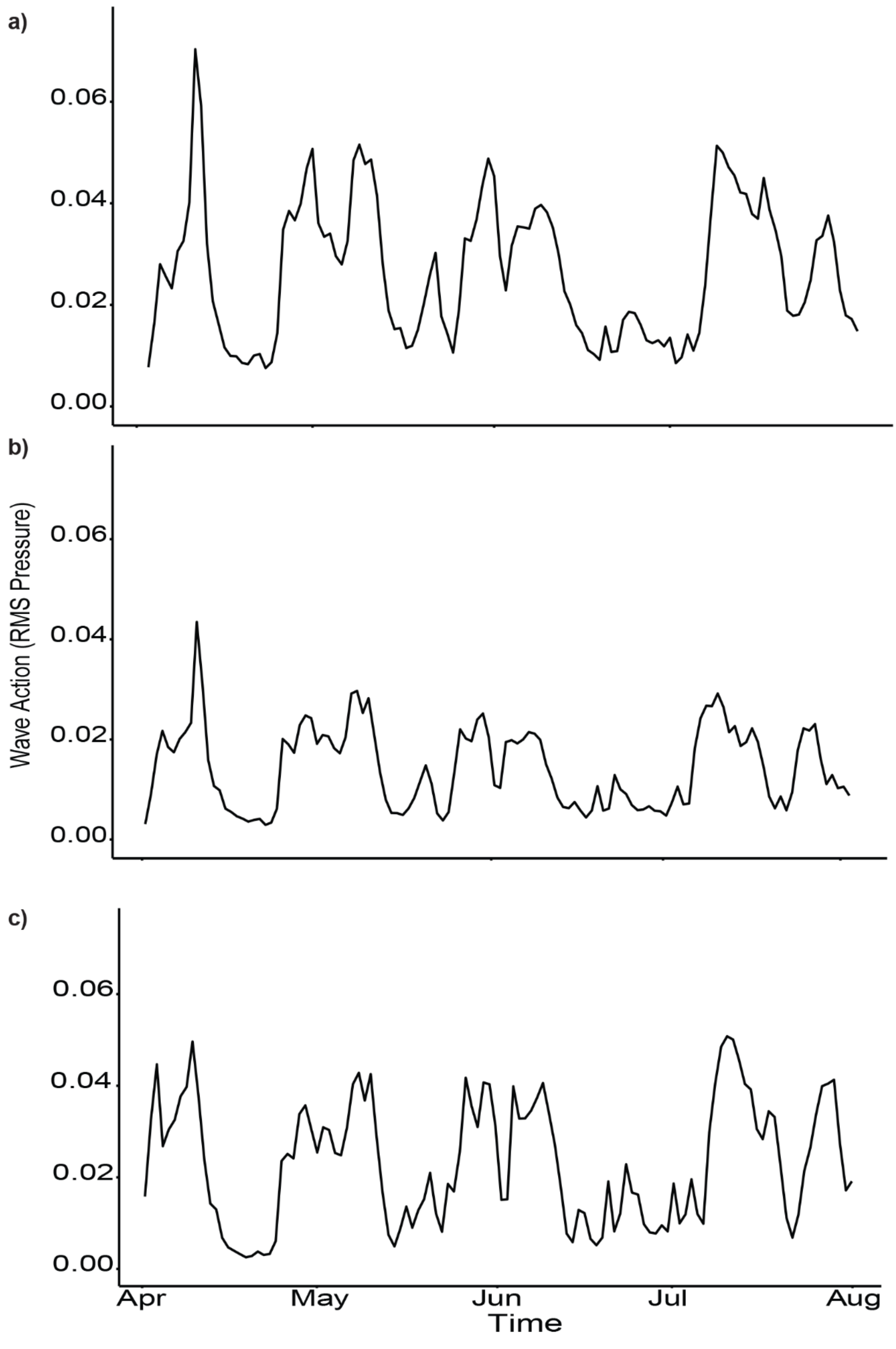

Figure S3: The daily average wave action recorded at a) Bay Rock Reef, b) Middle Reef, and c) Rattlesnake Island Reef.

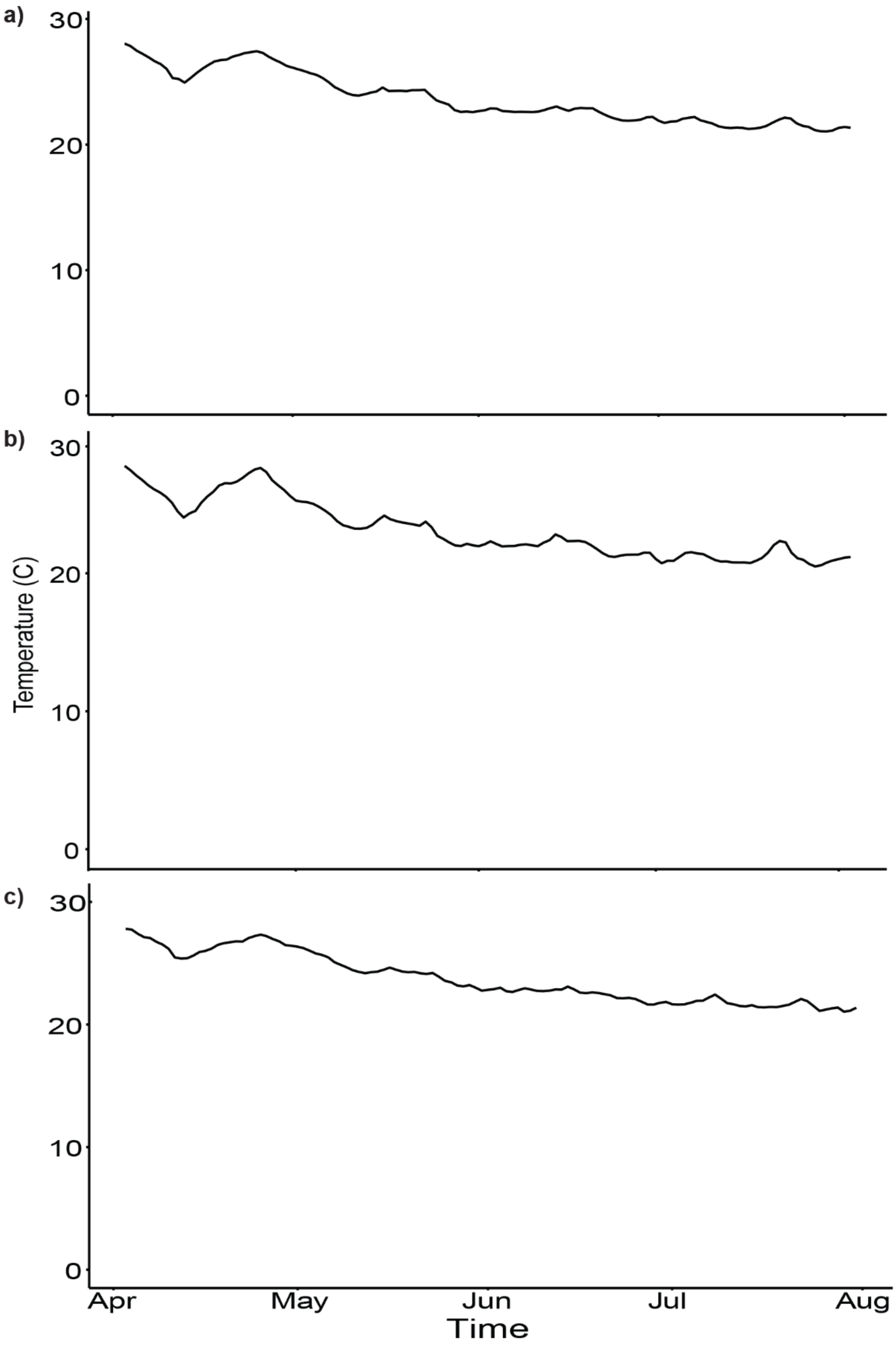

Figure S4: The daily average temperature recorded at a) Bay Rock Reef, b) Middle Reef, and c) Rattlesnake Island Reef.

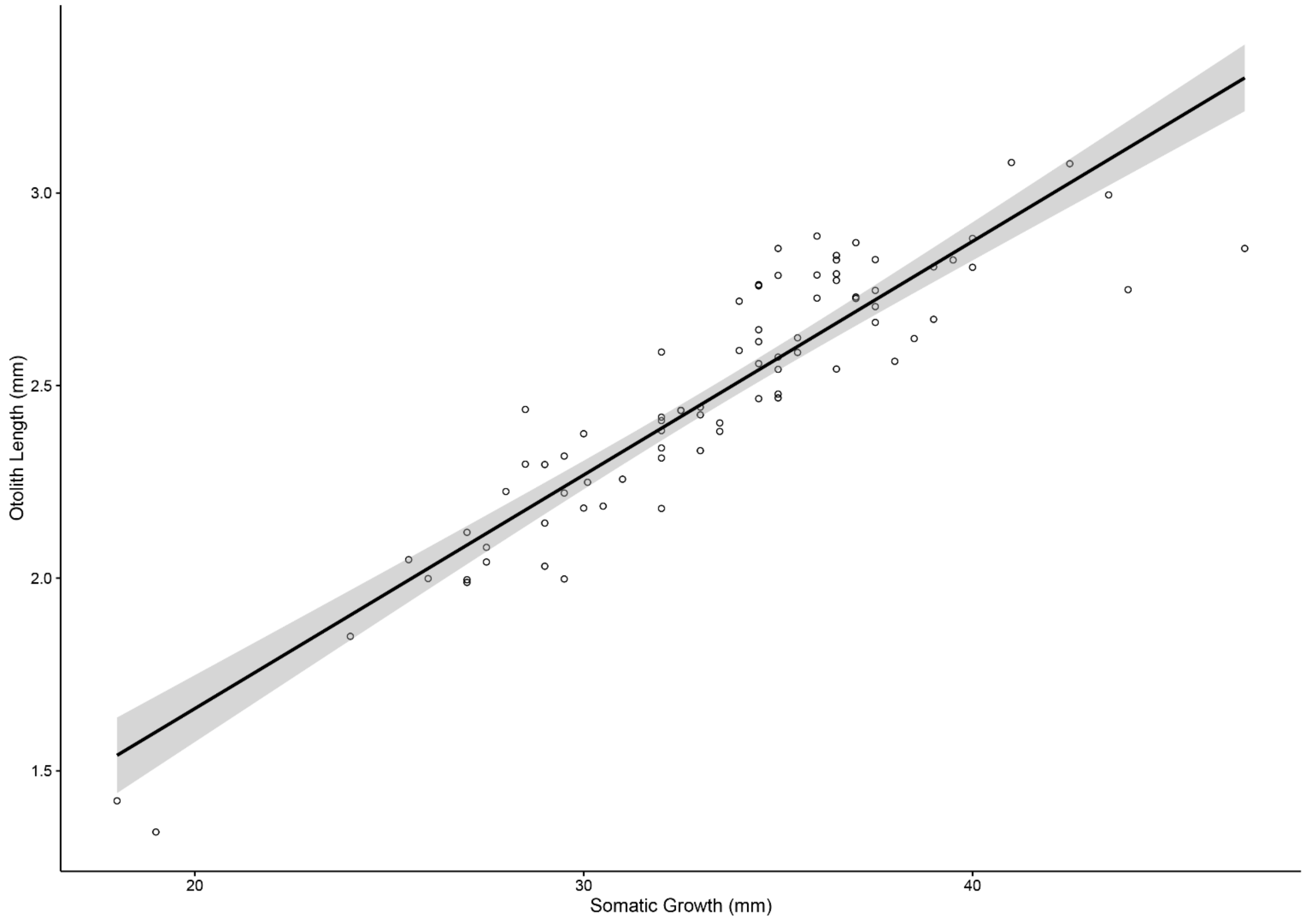

Figure S5: Correlation between somatic growth, measured as standard length, and otolith length.

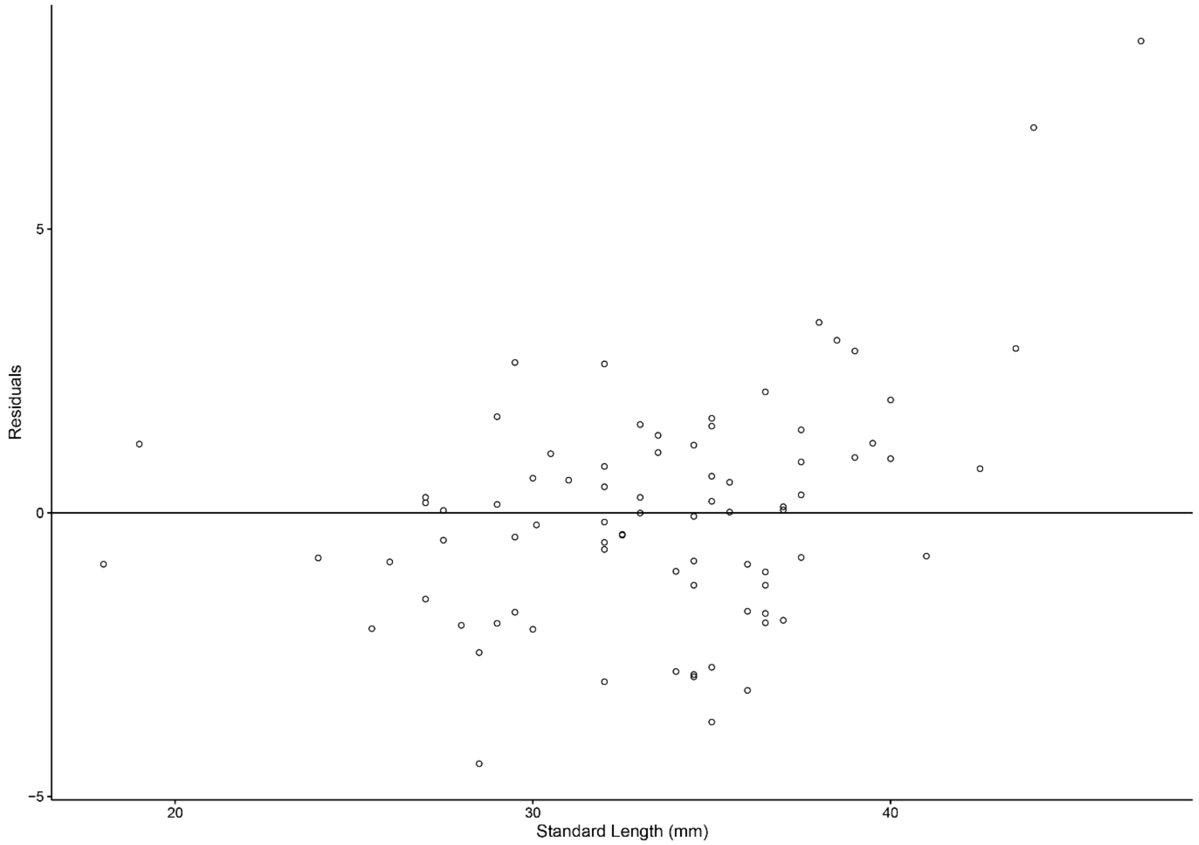

Figure S6: The residuals of the regression model for standard length and otolith length.

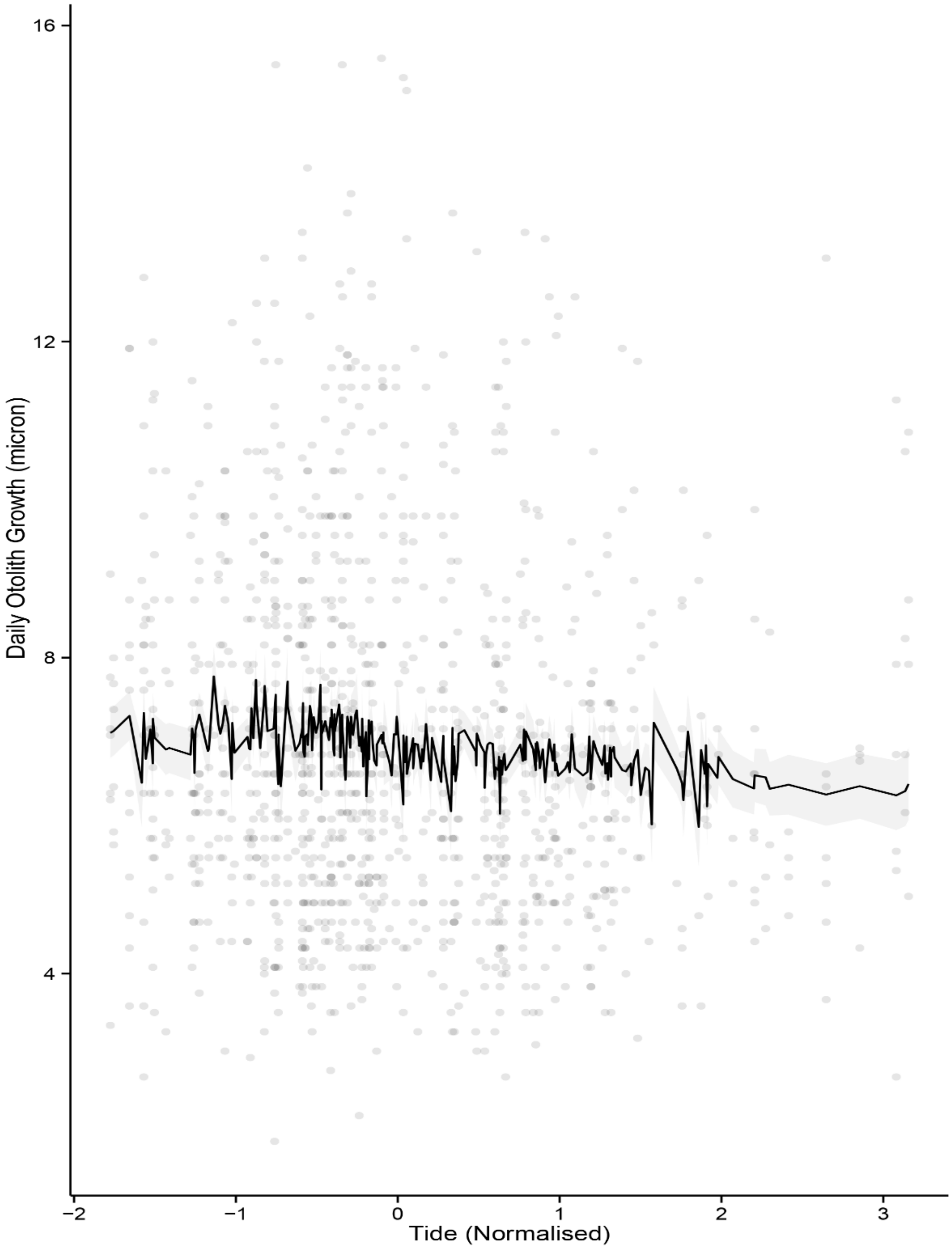

Figure S7: The linear mixed effects model predicted fit of the relationship between normalized tidal range and daily otolith increment width. Grey shading around the black line represent bootstrapped 95% confidence intervals. Grey dots represent the raw data.

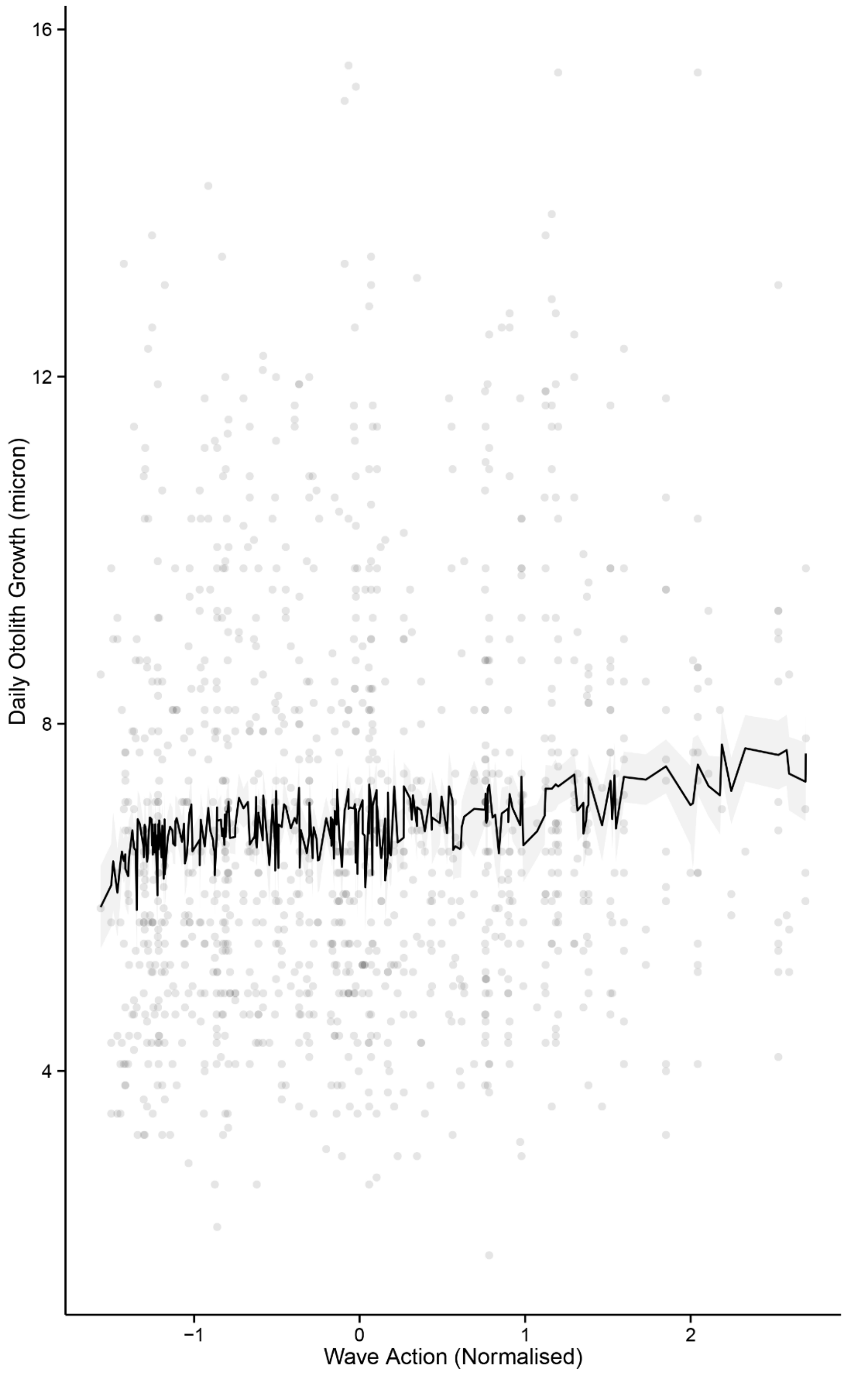

Figure S8: The linear mixed effects model predicted fit of the relationship between normalized wave action and daily otolith increment width. Grey shading around the black line represent bootstrapped 95% confidence intervals. Grey dots represent the raw data.

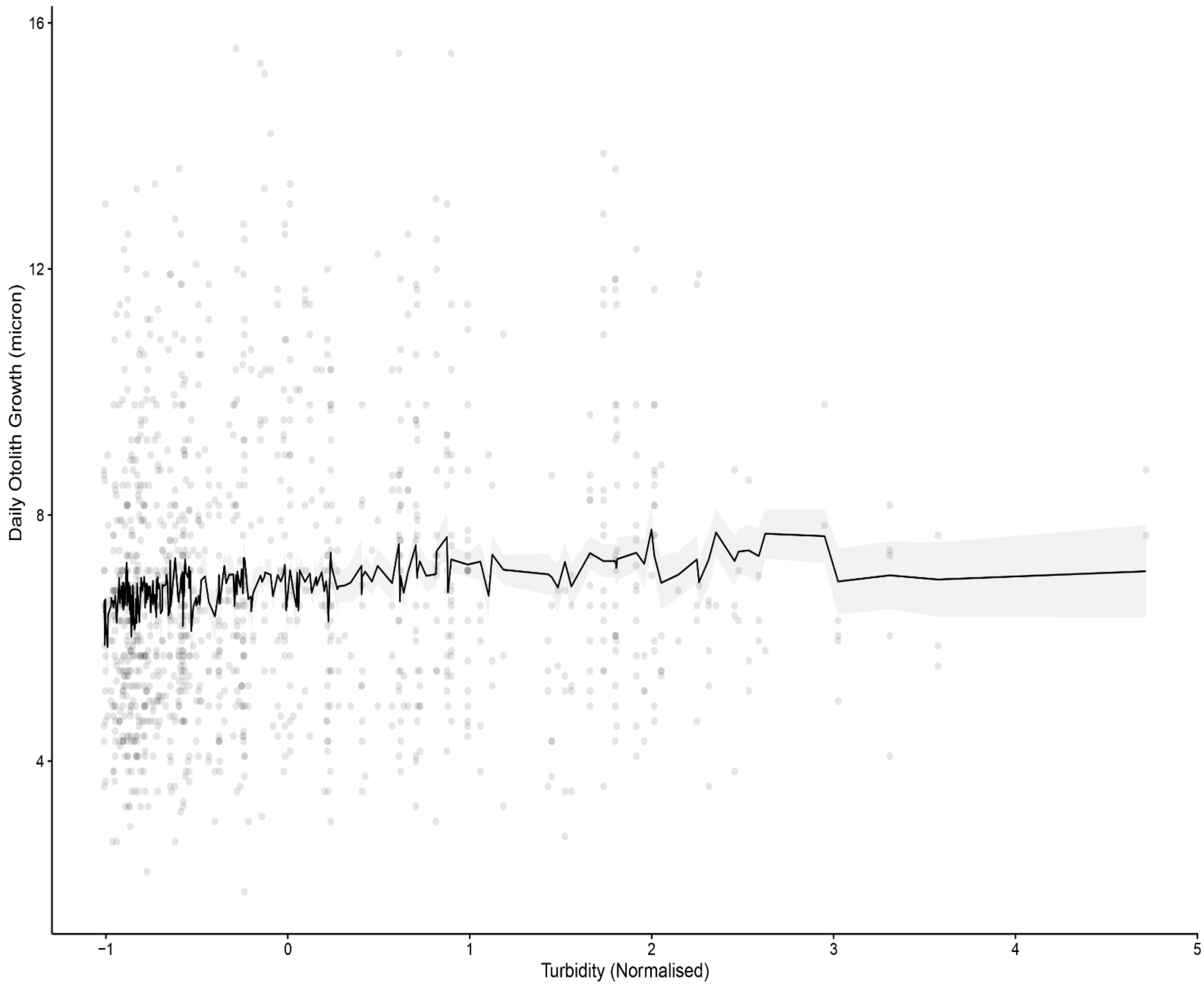

Figure S9: The linear mixed effects model predicted fit of the relationship between normalized turbidity and daily otolith increment width. Grey shading around the black line represent bootstrapped 95% confidence intervals. Grey dots represent the raw data.
